# Emerging Medical Technologies and Their Use in Bionic Repair and Human Augmentation

**DOI:** 10.3390/bioengineering11070695

**Published:** 2024-07-09

**Authors:** Albert Manero, Viviana Rivera, Qiushi Fu, Jonathan D. Schwartzman, Hannah Prock-Gibbs, Neel Shah, Deep Gandhi, Evan White, Kaitlyn E. Crawford, Melanie J. Coathup

**Affiliations:** 1 Limbitless Solutions, University of Central Florida, 12703 Research Parkway, Suite 100, Orlando, FL 32826, USAviviana.rivera@ucf.edu (V.R.); 2 Biionix Cluster, University of Central Florida, Orlando, FL 32827, USA; qiushi.fu@ucf.edu (Q.F.); kcrawford@ucf.edu (K.E.C.); 3 Department of Mechanical and Aerospace Engineering, University of Central Florida, Orlando, FL 32816, USA; 4 College of Medicine, University of Central Florida, Orlando, FL 32827, USA; jo535595@ucf.edu (J.D.S.); hprock@knights.ucf.edu (H.P.-G.); neel.shah264@knights.ucf.edu (N.S.); deep.gandhi@knights.ucf.edu (D.G.); ev363752@ucf.edu (E.W.); 5 Department of Materials Science and Engineering, University of Central Florida, Orlando, FL 32816, USA

**Keywords:** neuroprosthetics, exoskeletons, biohacking, ethics, brain–machine interface, human augmentation

## Abstract

As both the proportion of older people and the length of life increases globally, a rise in age-related degenerative diseases, disability, and prolonged dependency is projected. However, more sophisticated biomedical materials, as well as an improved understanding of human disease, is forecast to revolutionize the diagnosis and treatment of conditions ranging from osteoarthritis to Alzheimer’s disease as well as impact disease prevention. Another, albeit quieter, revolution is also taking place within society: human augmentation. In this context, humans seek to improve themselves, metamorphosing through self-discipline or more recently, through use of emerging medical technologies, with the goal of transcending aging and mortality. In this review, and in the pursuit of improved medical care following aging, disease, disability, or injury, we first highlight cutting-edge and emerging materials-based neuroprosthetic technologies designed to restore limb or organ function. We highlight the potential for these technologies to be utilized to augment human performance beyond the range of natural performance. We discuss and explore the growing social movement of human augmentation and the idea that it is possible and desirable to use emerging technologies to push the boundaries of what it means to be a healthy human into the realm of superhuman performance and intelligence. This potential future capability is contrasted with limitations in the right-to-repair legislation, which may create challenges for patients. Now is the time for continued discussion of the ethical strategies for research, implementation, and long-term device sustainability or repair.

## 1. Introduction

As both the proportion of older people and the length of life increases globally, a rise in degenerative diseases, disability, and prolonged dependency is projected. As a result, scientific innovation has prompted the Bio-Implants market to flourish (10.1% Compound Annual Growth Rate with a forecast of >USD 134 billion by 2024) with new discoveries, improvements, and advancements in both implantable and wearable devices [1]. The goal is to ensure longer periods of good health, a sustained sense of well-being, with extended periods of activity, social engagement, and productivity. In principle, existing and emerging materials-based technologies will offer the possibility of reducing medical illness and injury, thereby extending life expectancy and improving quality of life for both the young and old. This will allow people to remain healthy and independent well into old age, by not only adding more years to their lives, but also, by adding more life to their years [2]. Further, the increased awareness of new technology is causing a rise in the number of younger patients not only opting for surgery, but with many looking for preventative rather than curative care and ranging from various electronic to mobile devices [3]. However, such devices can have complex mental health effects, including altering psychosocial development, self-perception for the wearer, and social stigma. An arguably surprising phenomenon involves those who are using modern technology to attain greater levels of physical and sensory mastery, with the purpose of actively creating new physiological capabilities and functions that are otherwise non-existent in the normal healthy human. Creating seamless technology-driven environments for human interaction is plausible, but what happens when man merges with machine? 

This emerging area of inquiry not only drives research towards improved preventative and restorative medical care, including novel implants to remote medical monitoring, but also makes possible the expansion and improvement of everyday human skills, actions, senses, and cognition. Biohacking is a broad term that has at least two meanings. First, is the practice of changing the body’s chemistry and/or physiology. This includes simple dietary changes, listening to music, and taking supplements, to targeted changes to the gut microbiome, gene therapy, or methods to modify genetic or brain function to improve oneself (being faster, stronger, mitigating a predisposition for a disease, better focus, memory, energy etc.). Second, and a more extreme direction of biohacking, involves using bionic devices to extend or improve human capabilities or to augment human function. The synergy of cybernetics, biopunk (a subgenre of science fiction that focuses on biotechnology and synthetic biology), and citizen science (the collection and analysis of data relating to the natural world by members of the general public) has led to the formation of a media-activist community. As such, many contemporary technological discoveries are focused towards developing both permanent and temporary methods to be worn, implanted, or ingested with the goal of replacing or augmenting human performance and intelligence beyond their normal limits (e.g., the use of brain–machine interface devices, and genetic engineering). While many scientific research investigations are being carried out within traditional institutions globally, the “do-it-yourself” citizen science of integrating body modification with technology is rapidly emerging. Presently, bioelectronic devices are widely used in every facet of human daily activities. 

Epidermal electronics, biosensors, and artificial intelligence are quickly evolving within ever expanding healthcare technologies. For example, the use of a breadth of devices including cosmetic, fitness, biomonitoring, entertainment, and physical performance means that the human–machine interaction is increasingly becoming more normalized where both implantable and wearable technologies have increasingly become accepted. Thus, the opportunities for technological application to both healthcare and human augmentation abound. However, these advances have introduced the need for further conversations on the ethics surrounding their implementation. For example, navigating ethical use is at times unclear. In particular, the concept of embracing human augmentation, commonly referred to as transhumanism, presents many ethical and philosophical questions. Therefore, this review examines emerging, smart “bionic” materials, devices, neuroprosthetic technologies, and genetic engineering research developed to advance medical treatment, and how their uses may also benefit human augmentation. Additionally, the review explores the growing social movement and ethics of transhumanism, considering the possibilities and challenges to use implant technology to push the boundaries of what it means to be a healthy human into the realm of superhuman performance and intelligence. This review is not intended to be exhaustive, but rather a summary that highlights recent progress in each of these emerging areas. 

## 2. Smart Neuroprosthetic Devices: Restoring Sensory and Motor Functions Following Injury and Limb Loss

**Figure 1:**
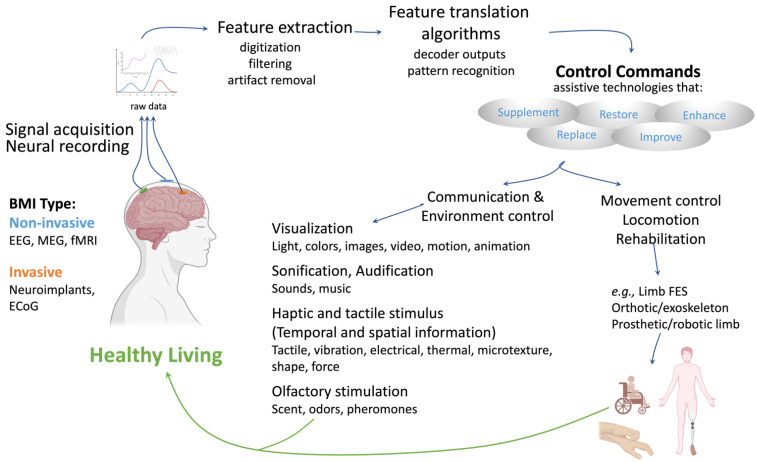
Brain–machine interface (BMI) design and operation. Electrical or other signals reflecting brain activity are recorded from the scalp, the cortical surface, or within the brain. Magnetoencephalography (MEG) detects magnetic fields created as individual neurons “fire” within the brain, pinpointing the active region within a millimeter and can follow the movement of brain activity as it travels from region to region. Functional magnetic resonance imaging (fMRI) exploits the changes in the magnetic properties of hemoglobin as it carries oxygen. Activation of a part of the brain can increase the ratio of oxyhemoglobin to deoxyhemoglobin. In a similar way to non-invasive EEG, electrocorticography (ECoG) detects and measures the electrical activity of the brain; however, ECoG measurements are taken following direct electrode contact with the cortical surface of the skull. Thus, ECoG has become a tool for detecting brain activity with higher-quality signals to EEG [11]. These signals are analyzed to measure signal features (e.g., single neuron firing rates, amplitudes of EEG rhythms) before their translation into commands that operate applications to replace, restore, enhance, supplement, or improve natural central nervous system outputs [12]. Many commercial ECoG electrode arrays are used clinically and differ in their shape, number of electrodes, spacing, thickness, and materials used. In recent clinical fields, ECoG electrodes are generally implemented for invasive extra-operative monitoring in, for example, patients with drug-resistant epilepsy, and in identifying precise seizure onset zones for resective surgery [13,14]. ECoG’s feasibility is also increasingly being used for rehabilitation purposes in patients with locked-in syndrome, and spinal cord injury [11].

The human brain and nervous system interact with the world through various sensory inputs that produce appropriate motor-controlled actions involving the arms, legs, and/or body in addition to hormone excretion. When the neural pathways between the brain, spinal cord, and these biological apparatuses are disrupted by disease (e.g., cancer) or traumatic injury (e.g., a road traffic accident), tissue damage can result in temporary or permanent changes in sensation, movement, strength, and bodily functions. Presently, it is reported that between 10 to 80 million individuals suffer from damage to or degeneration of the spinal cord each year [4]. One in 50 (5.4 million) people in the U.S are reported to live with some form of paralysis, and the associated annual healthcare costs are estimated to be USD 40.5 billion [5]. Neuroprosthetic technologies are designed to restore, enhance, supplement, or improve the impaired sensory and motor functions by restoring natural brain-to-body communication or through replacing the damaged sensory and motor organs using artificial devices, thereby completely bypassing the injured tissue interface (Figure 1). This is mostly achieved through use of multi-electrode technology that allows signals from the native nervous system to be decoded and used to control external devices such as prosthetics, cursors, or robots. Conversely, multi-electrode technology also allows the reverse; external signals can be delivered to the brain through native neural stimulation, for example, from cochlear implants for neural deafness, subthalamic implants for drug-refractory parkinsonism, and stimulation-based functional mapping of the cortex using subdural surface electrodes [6,7,8]. Brain–machine interface (BMI) devices hold the promise of achieving the ultimate prize, which is bridging interaction and function for patients and potentially regaining reliable and intuitive control when function has previously been lost due to disease or injury, or at the very least, partially restore muscle movement, vision, and other normal functions [9]. Patients who would benefit from a BMI can be stratified into three distinct groups: (1) those who have no useful neuromuscular control and are thus permanently “locked in”; (2) patients who retain a limited capacity for neuromuscular control; and (3) people who sustain a considerable capacity for neuromuscular control and can use muscle-based technology [7]. Although debate exists regarding locked-in syndrome and BMI resistance, studies show individuals may benefit if a BMI device is implicated before becoming entirely locked in. Patients with conditions such as amyotrophic lateral sclerosis, severe cerebral palsy, and brain stem strokes, would fall into category two, as these patients retain limited movement and minimal muscle function. Lastly, category (3) patients are those who have suffered from high spinal cord injuries. In this case, the BMIs rely on electromyography (EMG) from facial muscles or gaze.

Clinical applications of BMI can also be stratified according to its assistive tasks, such as locomotion, movement control, neurorehabilitation, communication, and environmental control. Locomotion applications focus on patient mobility, whereas movement control aims to address common motor control activities such as brushing teeth and holding towels. Electronic implants for the eyes, spinal cord, and brain are offering hope to people who are experiencing the debilitating effects of Parkinson’s disease, stroke, or macular degeneration. Equally, technology able to translate neural activity into speech would be transformative for people who are unable to communicate orally or through writing. A reported study in 2019 designed a neural decoder that leveraged kinematic and sound representations encoded in human cortical activity to synthesize audible speech [10]. Recurrent neural networks decoded directly recorded cortical activity into representations of articulatory movement, which were then transformed into speech acoustics. Using closed vocabulary tests, listeners could readily identify and transcribe speech synthesized from cortical activity, significantly advancing the clinical possibilities of using speech neuroprosthetic technology to restore spoken communication. Finally, environmental control BMIs target the ability to control environmental tasks, such as turning on the TV or lights, changing the temperature, and even turning the oven on and off. Neuroprosthetic integration with around home smart systems may provide new levels of independent living and quality of life as we stride into the future.

### 2.1. The Brain–Machine Interface

Brain–machine technology allows a human brain and an external device to bidirectionally communicate with one another (for a detailed review see [15]). For example, a BMI can record and decode motor signals from the motor cortex of a paralyzed human and use these signals to control artificial limbs or electrodes, thereby directly influencing motor output and bypassing the damaged and physiologically insufficient tissue within the injury site (i.e., peripheral nerves and muscles within the limb). This technology relies on our understanding of the neurophysiological principles of information representation, which is determined through analyzing the spatiotemporal patterns of neuronal activities. This includes identifying the tuning of a neuronal firing rate (i.e., the stimulus that evokes the greatest response) or the synchronization/desynchronization activity of ensembles of neurons in response to sensory and motor events. Most of the existing research has focused on the interpretation of neural signals, which are recorded from a large number of sensors. The decoding of these high-dimensional signals utilizes various statistical and machine learning algorithms to estimate the underlying information, such as movement or motor intent. In contrast, the sensory BMI is more difficult as our knowledge about how to effectively convert the flow of information from the external world into neural activity through stimulation of neurons remains limited.

The BMI can be invasive or non-invasive. The non-invasive approaches typically measure electrical activity generated from the brain tissue using multi-channel electrodes (i.e., electroencephalography) or the associated hemodynamic changes using functional near-infrared spectroscopy when placed on the skin over the skull [16,17]. Therefore, these interfaces do not require surgery to record neuronal signals and their use and performance has resulted in many successful applications [18,19] such as controlling the cursor on a computer [20], a prosthesis [21], exoskeletons [22,23,24], functional electric stimulation for hand opening and closing [25], and robots [26,27,28]. A particularly useful application allows patients with complete body paralysis the ability to communicate using spelling devices [29,30,31,32]. However, the major challenge for non-invasive approaches is the low signal-to-noise ratio due to the presence of various forms of noise and artifacts, and low spatial resolution. Therefore, non-invasive BMIs are limited to low-bandwidth communication, and the intuitive control of fine movement. Presently, the delivery of non-invasive sensory stimuli is very difficult if not impossible [26]. Recent advances within the field have begun to investigate the implications of using non-invasive techniques for the stimulation of deep brain structures [33]. Temporal interference (TI), which functions through the application of kHz-range electrical fields of multiple and varying frequencies, allows for the targeting of specific brain regions with minimal exposure to adjacent non-targeted brain structures. Remarkably, Grossman et al. [34] used non-invasive TI stimulation to the motor cortex region of the brain to activate neurons and drive evoked movements in mice. Through altering the currents delivered to a set of immobile electrodes, the authors reported steerable and tuneable alterations in the movement patterns of the whiskers, forepaws, and ears. In particular, targeted specificity in neural stimulation using TI holds potential for the treatment of diseases linked to structural hyperactivity, such as Alzheimer’s disease, schizophrenia, and temporal lobe epilepsy. However, future pursuits in stimulating motor responses and function to the limbs in humans must first overcome the challenges of the prohibitive and high injection currents needed when considering the size differences between humans and mice [35].

In contrast, invasive approaches provide bidirectional access to local neural activities using intracranial electrode arrays. These arrays have been used for patients with tetraplegia to control highly articulated robotic limbs [36,37], through using functional electrical stimulation of the native limb to perform functional reach-and-grasp tasks [37,38], or to provide sensations of touch through intracortical microstimulation [39]. Recently, highly complex motor behavior was decoded from cortical neural signals. Herein, a patient paralyzed due to a spinal cord injury was able to use an intra-cortically implanted system to produce a typing speed of 90 characters per minute with greater than 94% accuracy via an imagined handwriting movement [40]. Though spinal cord injuries often result in damage to neurological function and subsequent impairment of the generation of movement, recent work in the field has considered the usage of spinal cord electrical stimulation to recover functionality. Remarkably, Lorach et al. [41] restored communication between the brain and spinal cord using a wireless digital bridge that enabled an individual with chronic tetraplegia to stand and walk naturally and autonomously on complex terrains and after paralysis due to spinal cord injury. The technology utilized a brain–spine interface (BSI) which consisted of fully implanted recording and stimulation systems. Neurosurgical implantation of the device involved the insertion of an electrode array onto the sensorimotor cortex of the brain and implantation of a pulse generator into the spinal cord, allowing for the activation of target muscles towards ambulation. Stimulation provided by the BSI allowed for the recovery of lower extremity movement, enabling aided and unaided walking upon electrical stimulation. Further, rehabilitation of neurological control also allowed for autonomous movement when the BSI was no longer generating an electric current, and instead, the participant was only aided by crutches. In the case of cervical spinal cord injuries, symptoms present predominantly in the loss of function of arm and hand impairment. The ARC^EX^ device functions through the application of electrical stimulation via surface electrodes to the spinal cord, completed through stimulating sensory neuronal fibers [42]. In conjunction with rehabilitative therapy, the ARC^EX^ device has been shown to be capable of recovering quality of life, motor, and sensory capabilities when investigated in sixty study participants.

Loss of speech following paralysis significantly reduces quality of life and decoding speech from neural activity is highly challenging. Recent discoveries have developed high-spatiotemporal-resolution neural interfaces together with the adaptation of state-of-the-art speech computational algorithms that have resulted in rapid and impactful advancements in decoding neural activity into text, audible speech, and facial movements [43]. Thus, speech brain–computer interfaces (BCI’s) have the potential to restore communication. Audible speech acoustics can be directly synthesized from cortical activity by leveraging neural representations of vocal tract articulator kinematics [10]. Using closed vocabulary testing techniques, the participants readily identified and transcribed speech that was synthesized from cortical activity. Further, Willett et al. [44] developed a speech-to-text BCI that recorded spiking activity from intracortical microelectrode arrays and demonstrated that in a patient who could no longer speak due to amyotrophic lateral sclerosis, 2.7 times fewer errors (a 9.8% word rate) on a 50-word vocabulary, and a 23.8% word error rate on a 125,000-word vocabulary was achieved. This represented the first large-vocabulary decoding reported to date. Notably, the participant’s speech was decoded at 62 words per minute, and 3.4 times faster than previously recorded by a device, thus approaching the speed of natural conversation. The authors reported that the central premotor cortex contains a rich, intermixed representation of speech articulators even within a small area of tissue (3.2 × 3.2 mm^2^), thereby facilitating the improvements reported. Nevertheless, the efficacy of high-performance speech decoding has yet to be demonstrated in an individual that is fully locked-in with negligible residual motor function. Other important and challenging medical conditions where future investigations may accelerate the field of speech decoding are in examining its targeted use towards cortical forms of dysarthria, apraxia of speech, and aphasias [43]. 

To provide a reliable and long-lasting BMI using implantable electronic systems in soft brain tissues, multiple challenges remain before the widespread adoption of such systems clinically [45,46]. Challenges such as biocompatibility, miniaturization, scalability, and wireless communication must first be addressed. It is generally believed that increasing the channel count of a BMI could provide better spatial resolution, larger volume coverage, and higher reliability during recording. Most existing functional BMIs implanted in humans have hundreds of channels, but it is desirable to have thousands or even more channels in future devices. While some progress has been made to meet such demand with large-scale electrode arrays [47,48,49] and distributed sensor networks [50,51,52], further investigations will be needed to determine whether existing neural decoding algorithms can be scaled efficiently to match the hardware advancement. For example, significant progress has recently been made in the development of Neuralink, a unique system that combines ultrafine, flexible polymer probes, a neurosurgical robot, and high-density miniaturized custom electronics, offering channel magnitudes that surpass existing BMI technology [47]. However, increasing the channel count is also associated with increased interface contact area and presents new challenges to biocompatibility such as suppression of immunological reactions and minimizing the displacement of brain tissue, which are currently being addressed with innovative materials, structures, and surgical technologies [53]. Similarly, Neuropixels, which are characterized by their high channel and density, are silicon-based probes that record electrical signals beyond the capabilities of current clinically approved devices. By increasing the density of signal production, electrode size may be decreased, allowing for a better integrated implementation amongst a high quantity of recording chips. Following safety, sterilization, and electrical noise mitigation in the intraoperative setting, such probes were capable of recording activity abnormalities within single neurons to gather data following their first-in-human use post-craniotomy, and for a variety of indications [54], and during resection surgery for epilepsy or tumors and deep brain stimulation electrode placement in patients with Parkinson’s disease [55]. Notably, ongoing research pursuits also target the use of single neuronal activity in restoring communication and motor control in patients suffering from traumatic neurological injuries [56]. Further, advancements towards the technology behind high-density electrodes also provide potential towards the improved understanding of neurological pathologies.

The field of brain–computer interfaces is poised to advance from the traditional goal of controlling prosthetic devices using brain signals to combining neural decoding and encoding within a single neuroprosthetic device. Such a device could act as a “co-processor” for the brain, with applications ranging from inducing Hebbian plasticity for rehabilitation after brain injury to reanimating paralyzed limbs and even enhancing memory. While mapping of the brain’s complex distribution of functions and linking them to human thought or intentionality remains complex, this technology, if successful, may lead to even deeper integration with electromechanical systems. This, in turn, may offer many advantageous and novel medical applications including the ability to seamlessly improve health and restore function following human ailment, while also playing a considerable role in augmenting human performance. In summary, the vast possible clinical applications of BMI technology are exciting, considering its potential restorative capabilities. However, this also raises the question of whether the implementation of advanced BMIs will ultimately deliver the anticipated gains in human augmentation, possibly resulting in a growing divide in human physical performance and cognition for those who use it.

### 2.2. Prosthetic Limbs and Peripheral Neural Interfaces

There are approximately 2.1 million people living with limb loss in the United States and due to our aging population and a projected increase in illness and injury, this number is anticipated to double by 2050 [57]. Approximately 185,000 people undergo amputation surgery each year, with 300–500 of these surgeries being performed each day, representing 1 amputation performed every 30 s, globally [58]. The main reason for limb removal is due to vascular disease (54%), including diabetes and peripheral arterial disease, trauma (45%), and finally, cancer (<2%), with hospital costs estimated at more than USD 8.3 billion per year [57,58]. It has been reported that 30% of people with limb loss experience depression, anxiety [59], and emotional stress [60], which can lead to social isolation, relationship breakdown, alcohol dependence [61], poor long-term quality of life, and reliance on pain medication [62]. For this reason, a prosthesis plays an important role in rehabilitation, helping to restore a large part of patient mobility and contributes to overall independence. 

Using artificial limbs to restore human motor function dates back as early as the ancient Egyptians. From the early cosmetic devices, the electromechanical design of artificial limbs has advanced tremendously. The most critical aspects of any prosthesis are the quality of the interface between the limb remnant and the artificial prosthesis, human dexterity, durability, and cosmetics [63]. As such, bioengineers are increasingly interested in creating human–machine interfaces embodied by a prosthetic limb so that it feels like an extension of the body. Earlier versions of prosthetic limbs were mostly passive or powered by human body movement, providing limited support of body weight or simple grasp functions, for example. However, as technology advanced, modern prostheses are actuated based on signals acquired from the peripheral neuromuscular system, thereby achieving enhanced flexibility and dexterity [64,65]. Similar to the BMI technologies, the interface between the human user and the artificial limb can be invasive or non-invasive. The non-invasive systems are wearable and measure the electrical or mechanical signals from residual muscle contractions at the surface of skin using surface electromyography [66], force myography [67], or ultrasound imaging [68]. These approaches are simple to implement, but their function is also inherently limited by the low signal-to-noise ratio due to variability at the sensor–skin interface [69] and by the dependence on the volume of residual muscles [70,71], therefore preventing natural and intuitive human–machine integration. Several invasive approaches have been developed to address these challenges. Implantable sensors, such as intramuscular wireless electrodes [72] and magnetic sensors [73,74], have been developed to obtain muscle contraction signals with higher reliability and specificity. However, they still depend on the availability of the residual muscles. The surgical technique known as targeted muscle reinnervation (TMR) can be used to redirect neural signals from the missing muscle to a new site (i.e., the use of “spare” muscles) [75]. This technique transfers nerves that have lost their original innervation (e.g., the median nerve that innervates the hand muscles) to a different muscle (e.g., the chest muscles of patients with a shoulder disarticulation amputation) [76]. With time, TMR leads to reinnervation and hyper-reinnervation of the targeted muscle, enabling more surface recording sites in patients with a high-level amputation [77].

Efferent neural commands for muscle contraction can also be directly recorded at the nerve level using peripheral nerve electrodes [78]. Peripheral nerves consist of perineurium-encapsulated axon groups (i.e., fascicles), and the electrodes can be positioned with respect to the peripheral nerve structure in different ways. Extraneural electrodes, also referred to as cuff electrodes, are commonly used as they are less invasive because they avoid the foreign biological responses that come along with penetrating electrode contacts [79]. In contrast, penetrating electrodes, such as inter-fasicular electrodes [80], intra-fasicular electrodes [81], and regenerative electrodes [82,83], can achieve better spatial resolution and specificity while still facing challenges of longevity, stability, and infection. A major advantage in the use of peripheral nerve interfaces is the ability to directly stimulate the afferent axons that provide natural sensory feedback. As humans can accurately sense the position, speed, and torque of their body parts [84], this proprioceptive characteristic contributes a critical role for enhancing the embodiment of the artificial limb. To this end, clinical studies have shown that dexterous use and cognitive integration of a prosthetic limb can be significantly improved when cutaneous and proprioceptive feedback is provided through peripheral nerve stimulation [85,86,87,88,89]. Recently, Clites et al. [84] developed an alternative to provide proprioceptive sensory feedback without implanted electronics. The agonist–antagonist myoneural interface (AMI) surgically connects an agonist and an antagonist muscle in series so that the contraction of one muscle stretches the other; therefore, preserving dynamic muscle relationships that exist within the native anatomy. The closed-loop joint torque control in AMI patients displayed overall improved control over the prosthesis with more natural reflexive behaviors during stair ambulation. The authors concluded that the results provide a framework for integrating bionic systems with human physiology. Another invasive approach that enhances integration between the user and prosthetic limb is osseointegration surgery [90]. One major challenge involving traditional prosthetic limb wearing is the use of a socket mount, which leads to frequent fitting and stability issues due to the mechanical interaction between the socket and residual limb [91,92]. Osseointegration surgery directly anchors a metal implant into the residual bone to support a prosthetic limb attached percutaneously. Such a procedure not only eliminates the various issues associated with socket mounts and provides improved comfort, but also dramatically improves functional performance [93,94,95,96], promotes osseoperception [97], and the “part of me” embodiment experience [98]. As for most implantable devices, the osseointegration procedure also faces challenges involving infection. Additionally, periprosthetic fracture and other mechanical complications are also reported [99]. As such, these issues need to be addressed through future improvements of the surgical technique and implant design [100]. 

Functionally, the use of neuroprosthetics has substantially impacted, and improved patient quality of life. Activities of daily living such as cooking, cleaning, eating, and housework can be performed without assistance from most users. Clinically, significant psychological improvements are reported in patients as well. Graczyk and colleagues [101] found when amputees used home-neural connected sensory prostheses, their quality of life, self-efficacy, self-image, and embodiment improved, and their perception of disability decreased. These examples clearly indicate that technological advances, such as neuroprosthetics, allow amputees to reconnect positively in their world. With increasing connectivity for internet of things devices (IoT), neuroprosthetic devices hold promise in providing alternative engagement methods for users, reenabling lost functionality and potentially gaining augmented functionality. To this end, data suggest that mechanically driven technology designed to restore function following disease or injury can also be applied to enhance human performance beyond the range of natural performance. For example, carbon fiber prosthetic legs, called blades [102], have been developed for amputee sprinters. However, scientific analyses have demonstrated that blades significantly augment running performance through enhancing an individual’s running efficiency. This is reported to be due to the blades being lighter in weight than the lower limbs they replace, requiring 20% less force to achieve running speeds, and as a result, can move 15% faster than the highest performance of sprinters with native, residual, and intact limb function [103,104]. Nevertheless, for a prosthetic limb to function seamlessly during human performance, sensory feedback is necessary. Future feedback may be achieved using force sensors, gyroscopes, and accelerometers that decode the user’s intent in real time, using sensory-responsive synthetic electronic skin that surrounds a prosthetic limb, or via sensory feedback from a light-sensitive neuroprosthetic in the case of an artificial retina. These examples, together with exoskeletons, are highlighted in the sections below. In each case, sensory feedback into the sensory cortices via a BMI is necessary for bidirectional information flow and enhanced sensorimotor performance, function, and learning [105].

### 2.3. Exoskeletons and Exosuits

**Figure 2:**
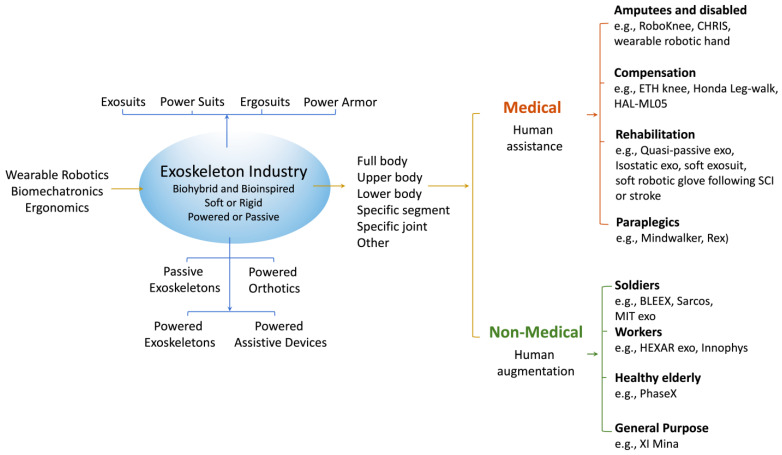
Classification of exoskeletons. Recent research has focused on load augmentation for soldiers/workers, assisting trauma patients, paraplegics, spinal cord injured (SCI) persons and for rehabilitation purposes. For medical exoskeletons, the motion trajectories for individual joints cannot be provided by the wearer as the patient cannot make the required movements. Thus, user interfaces, control strategies, mechanical interfaces, etc., need to be designed specifically to cater for the individualistic needs of the patient. For non-medical exoskeleton applications, the methods for measuring “user intention” are most important; therefore, facilitating actuated mechanisms that support the user’s actions and thus ensuring the desired motions are as natural as possible, is the key objective. HAL: Hybrid Assistive Leg, CHRIS: Cybernetic Human–Robot Interface System, BLEEX: Berkeley Lower Extremity Exoskeleton, MIT: Massachusetts Institute of Technology, SCI: spinal cord injury [108,128,129,130,132,133,141,142,143,144,145,146,147,148,149,150,151,152,153,154,155,156,157].

Exoskeletons are wearable robotic devices that are typically attached to the extremities of the human body improving mobility [106] and dexterity [107]. For disabled individuals, such as stroke survivors and patients with spinal cord injury, exoskeletons assist by overcoming the neuromuscular constraints and restoring motor ability [108,109,110,111]. For healthy individuals, exoskeletons may enhance strength, endurance, heavy lifting, and speed beyond their standard capacity [112,113,114,115,116] (Figure 2). An interesting extension of the exoskeleton is the development of artificial supernumerary limbs which are wearable robots that function alongside existing limbs to provide support for human activities [117].

The main consideration when designing exoskeletons is to minimize the extent to which the device may interfere with the users’ existing motor capacity, such as joint range of motion, metabolic energy expenditure, or normal movement patterns. Progress has been made in several engineering technologies to address this challenge. For actuation and mechanical design, electric motors paired with rigid articulations are the most common choice for high precision control, but they come with large inertia, and rigid contact between the device and body [118]. Alternative approaches have been developed to overcome these drawbacks. For example, hydraulic actuation can be used for applications that involve heavy loads due to its high-power-to-mass ratio [119,120,121]. The Ekso GT exoskeleton is one such example which has gained FDA approval for patients with hemiplegia due to stroke, various spinal cord injuries, and multiple sclerosis [122]. This exoskeleton utilizes materials such as aluminum, carbon fiber, and titanium to support patient weight and stability. Carbon fiber, known for its high strength-to-weight ratio, is used in areas including the feet, shins, and back to improve maneuverability and to facilitate normal movement patterns while remaining lightweight. The upper extremity exoskeleton primarily consists of titanium to increase durability and provide longevity to components under extended use. Further, sensors and control systems located within the braces in various locations in the exoskeleton quickly respond to user muscular activity by altering the output of the hydraulic power system. This design more readily conforms to body shape and allows for smoother and safer mobility, along with the facilitation of physical movement to reduce muscle atrophy and improve strength. Ameliorating the poor fit and increased risk of pressure sores seen in older exoskeleton models reduces challenges that primarily affect older individuals [123]. Together, this facilitates a more intuitive movement experience and response to kinetic gait changes [124]. Despite these advancements, the Ekso GT exoskeleton, as well as other prominent exoskeletons, remain limited by two non-mechanistic factors: high cost and training. As such, these exoskeletons are primarily housed at rehabilitation facilities with trained staff, limiting their broader usability to only individuals with the time and means of transport to attend the facility [125]. Nevertheless, in a randomized controlled trial, Edwards et al. [126] reported improvements in the clinical ambulatory status in patients with chronic incomplete spinal cord injury after 12 weeks, and 36 sessions of exoskeleton training. However, and notably, large gains in ambulation were also associated with upper and lower extremity musculoskeletal adverse events including orthopedic pain, neurological problems such as increased spasticity, and dermatological issues. In another randomized controlled trial, Rojek et al. [127] reported Esko GT-induced improvements in a greater number of recovery categories among ischemic stroke patients when compared to classic physiotherapy, noting that exoskeleton treatment was associated with a more beneficial response in balance and decreased unfavorable load transfer from the backfoot to the forefoot in gait mechanics.

Pneumatic actuation and cable transmission are used to reduce the rigidity and weight by creating “soft” exoskeletons [128,129,130,131]. Soft, lightweight, garment-like mechatronic devices forgo heavy, bulky components in favor of flexibility and versatility, increasing comfort, safety, and ease of use. Passive elastic components are added to reduce actuator power requirement and increase device compliance by regulating the flow of mechanical energy [132,133]. Several laboratories and manufacturers have developed iterations of these soft exosuits including the Harvard Biodesign Lab, the Shenzhen Institute of Advanced Technology, and HeroWear for specific consumer populations and functions [134,135,136]. For example, Apex 2, a trunk and back exosuit developed by HeroWear, was designed to reduce biomechanical loads applied to a user’s back, and primarily for use in the construction, agriculture, and manufacturing spaces. Notably, this design was developed for commercial use to augment human performance when lifting heavy loads. Though it utilizes materials such as aluminum and carbon fiber, the battery and hydraulic systems were replaced with springs and cables that transfer weight from loads on the hips, legs, and back rather than providing external power output. Adjustable straps, buckles, and nylon are used to provide a more secure fit that rests most of its weight on the hips rather than upper extremities. Thus, the Apex 2, and other similar exosuits, assist with lifting heavier loads and subsequently decrease the risk of lower back and hip injuries [136,137,138]. While HeroWear aims to reduce back strain and facilitate lifting movements, Harvard’s Biodesign Lab soft exosuit was designed to facilitate the precision and efficiency of normal human walking using wearable garments instead of the rigid materials typically used to fabricate exoskeletons. The advantages of this approach include unrestrained joints, decreased pressure points that can lead to the development of ulcers or other dermatologic phenomena, and a lightweight design more conducive to use in the elderly or in minorly disabled patients. By mimicking the underlying muscle and tendon biomechanics, these exosuits function in conjunction with a series of batteries and motors that use cables to transmit forces to joints [139]. Thus, this design offers assistance to individuals with minor gait impairments or nervous system damage that limits mobility but with otherwise physiologically functioning lower-limb musculoskeletal capabilities. Further, this approach has also been investigated in the upper extremity to treat neurological diseases such as amyotrophic lateral sclerosis. Indeed, in a small cohort of 10 individuals with amyotrophic lateral sclerosis and varying degrees of neuromuscular impairment, Proietti et al. [140] demonstrated exosuit-induced improvements in the active range of motion, functional activity (i.e., activities of daily living), and increased endurance, for example, in holding objects. Nevertheless, it is important to note that despite these promising endeavors, an exoskeleton versus an exosuit remains the only tool adept to treat patients with significant limitations in mobility and spinal cord injuries. Thus, the scope of exoskeleton usability continues to expand with developments in research and technology, paralleling to the breadth of patient populations for which this technology is intended.

However, a significant issue remains. How can we maximize the benefits of exoskeletons while allowing users to maintain effective control? As a device that must correctly follow and augment the user’s movement in diverse use scenarios, decoding the user’s intent in real time is not a trivial task. Various sensors have been used to provide information about the physical state of the human user, such as force and motion sensors, inertial measurement units, as well as invasive and non-invasive neural interfaces such as EMG and cortical implants. The challenge of decoding intent for such integrated human–machine systems is the need for accurate and fast interpretation of multi-joint movement. Spatial and temporal mismatch between the user’s own movement and the motion of the device may create discomfort or increase the user’s metabolic cost when using the device. However, and similar to the BMI and prosthesis control, the bandwidth of human–machine interfaces is still relatively limited with respect to the natural human motor capability that involves many muscles and joints. One approach meant to enhance the exoskeleton’s ability to anticipate the potential motor intent of the user, involves giving the exoskeleton context awareness through computer vision. This approach enables the exoskeleton to understand the environment and have predictive powers related to potential future motor tasks, therefore “sharing” the control effort [158,159,160]. This allows exoskeletons to have “real life” functionality. To this end, Zhang and colleagues [161] found their smart controller was able to estimate metabolic profiles and rapidly adjust torques for enhanced walking and running efficiency. Further, a technology that highlights the interplay between human and robot interaction is an active pelvis orthosis (APO), which is designed to support and stabilize the pelvis, lower back, and hips primarily during gait movements [162,163]. These devices are typically comprised of polypropylene polymers, aluminum, or steel for stability and durability, and foam paddings to support user comfort and long-term usability. Acting as a hybrid between the full exoskeletons and soft exosuits, these devices are actively powered, typically by batteries or other portable power sources to receive user input and module kinetic output via sensors placed around the device. These sensors typically include a combination of force sensors, gyroscopes, and accelerometers which may be part of the device or separately placed on the user. Recent advancements have seen the rise in the development of sophisticated control systems that utilize machine learning and artificial intelligence to more accurately measure movement patterns and thereby respond with greater precision [164]. D’Elia et al. [165] investigated the extent to which an APO interferes with the normal kinematics of human locomotion and found negligible interference in normal locomotion and minor relative displacements between device cuffs and body segments. Further, Monaco et al. [166] investigated the efficacy of a closed-loop APO design in terms of balance and postural control. The APO detected signs of balance loss using a novel control algorithm and found that the APO detected mechanical disruption in simulated falls in approximately 350 milliseconds, before applying counteracting torques at the hip to assist with balance recovery. Experimental analyses were conducted in eight elderly persons, and two transfemoral amputees with a moderate to severe risk of falls, and data show greater stability due to the APO behavior of “assisting when needed”. Continued advancements in sensory and machine-learning technologies in combination with durable and lightweight hardware components may prove useful in helping our aging population more safely complete activities of daily living. Future deployment of these technologies may indeed result in substantial decreases in healthcare expenditure.

Thus, the continuum of exoskeleton technologies provides expanded mobility opportunities for both non-disabled and disabled patients. The technology should continue to evolve and benefit from innovations in actuation, sensing, control, and neural interface technologies, and as we gain a better understanding of neuromuscular principles. It is predicted that future applications involved in augmenting human performance will be deeply influenced by the rise of computer-based systems or artificial intelligence, and that pursuing a mutually beneficial balance will be essential.

### 2.4. Electronic Skin

Human skin is a complex mechanical force-sensing organ that plays an important role in our daily interaction with the environment. Skin consists of an integrated and stretchable network of highly sophisticated sensors within a physical barrier. These sensors relay informational data about tactile and thermal stimuli to the brain and transduce information generated by physical contact into electrical signals and subsequently send it to the central nervous system for more complex processing. Efforts to create an artificial electronic skin (e-skin) with human-like sensory haptic capabilities were inspired by the discovery of new applications for this emerging area of science, particularly involving the possible application of flexible multi-sensory surfaces for use in autonomous artificial intelligence (e.g., robots), medical diagnostics, and biomimetic prosthetic devices capable of providing the same, if not a better, level of sensory perception than the organic equivalent [167]. Intelligent robots with large-area networks that provide skin sensing capabilities could extend their use to include highly interactive tasks, such as caring for the elderly [168] and sensor skins applied on or in the body could provide an unprecedented level of diagnostic and monitoring capabilities [169]. Current e-skins are already capable of providing augmented performance both in terms of superior spatial resolution and thermal sensitivity when compared to its natural organic equivalent. Serpentine and mesh structures have been adopted to achieve very high stretchability and softness, comparable to natural skin [170,171,172]. Different sensing capabilities have been developed by integrating tactile/pressure sensors [173], temperature sensors [170,171], strain sensors [170,174], humidity sensors [172,175], and chemical sensors [176], and by using advanced materials including single-crystal silicon, organic semiconductors, nanoparticles, nanowires, nanotubes, and graphene. Nevertheless, e-skin could be further improved through the application of targeted biological and biochemical sensing abilities. In recent years, researchers have made considerable advancements in enhancing the natural skin functions of sensation and physical protection. For example, pentacene-based organic field-effect transistors for pressure sensing, stretchable batteries, macroscale nanowire e-skin, self-healing e-skin, rechargeable stretchable batteries, and ultrathin stretchable organic photovoltaics have been investigated. These advancements allow for greater functionality when sensing pressure, temperature, strain, humidity, and chemicals [167]. Inspired by the wound healing capability of natural skin, other e-skin functionalities include its ability to self-heal and self-power the instrumentation.

Re-healable electronic skin is a recent development that is of significant scientific interest and the ability of e-skins to repair mechanical and electrical damage greatly expands its scope of utility. In 2018, Zou et al. [177] developed a fully recyclable and malleable e-skin containing tactile, temperature, and humidity sensors, using a novel dynamic covalent thermoset doped with silver nanoparticles. This polyamine polymer film rehealed following the application of heat and a solution composed of a mixture of terephthalaldehyde, diethylenetriamine, and tris (2-aminoethyl) amine in ethanol. While the traditional methods of bonding two materials typically rely on van der Waals forces, the use of heat-pressing together with application of the rehealing solution, allowed for the growth of oligomers and polymers that led to an e-skin connected through robust covalent bonding. The study showed that the skin can be rehealed following damage, such that it regained its mechanical and electrical properties comparable to the original e-skin. Similarly, a study by Tee et al. [178] developed a flexible and electrically conducting material that could sense mechanical forces and yet was able to self-heal repeatedly under ambient conditions. A composite material composed of a supramolecular organic polymer with embedded nickel nanostructured microparticles was created and displayed both mechanical and electrical self-healing capabilities where mechanical damage preferentially broke the large number of relatively weak hydrogen bonds, known to dynamically associate and dissociate at room temperature [179]. As a result, maintenance of the stronger covalent bonds allowed the e-skin to self-heal passively at the fractured site. On rupture, the initial conductivity was restored with an approximate 90% efficiency after 15 s healing time, and the mechanical properties completely restored after approximately 10 min. In 2017, Zhao et al. [180] developed a flexible e-skin with multifunctional sensing capabilities inspired by the thermosensation of the human sensory system. Using platinum sensing elements, the e-skin was capable of perceiving both mechanical and thermal stimuli, discriminating matter type and sensing wind using the thermosensation of a platinum ribbon array embedded on a polyimide substrate. The temperature of the array varied with conductive or convective heat transfer toward the surroundings, and external pressure stimuli were detected by measuring the elastic deformation of a porous elastomer covering on the heated platinum ribbon. When in contact with another substance, heat was transferred via conduction and through convection, a change in temperature allowed it to sense wind flow speed as well as the direction of airflow.

User-interactive e-skin is capable of spatially mapping touch via electrical readout and providing visual output as a human-readable response. Javey and colleagues first integrated thin-film transistors, pressure sensors, and organic light-emitting diode arrays to invent a device that provides an instant visual response to pressure and opened up a new era of user interactive e-skin [173]. Later, Chou et al. [181] reported on a stretchable e-skin with interactive color-changing and tactile sensing properties. Larson et al. [182] proposed an electroluminescent skin for optical signaling and tactile sensing. Despite these achievements, the above devices rely on an external power supply for both tactile sensing and touch visualization, which brings significant challenges to portability and integration. In 2020, Zhao et al. [183] took the concept of a triboelectric-optical model further and developed a self-powered, user interactive e-skin (SUE-skin) capable of simultaneously converting touch stimuli into electrical signals and real-time visible lights without relying on an external power source. SUE-skin, which is capable of interactive luminescence and tactile-sensing was able to achieve the conversion of touch stimuli into an electrical signal and instantaneous visible light at a pressure threshold of 20 kPa. By integrating the SUE-skin with a microcontroller, a programmable touch operation platform was built that recognized more than 156 interaction logics for easy control of consumer electronics. Zhang et al. [184] also mimicked the pressure-sensing behavior of biological skins by integrating multiple pressure-sensing modes and developing an e-skin that acts as a parallel-plate capacitor in order to detect and map tactile sensation. Inspired by the jellyfish, Atolla wyvillei, which produces bioluminescence in response to nociceptive stimuli, this e-skin technology incorporated an optical response to the detection of pressure. By combining the electrical and optical responses, a dual-mode response characteristic was developed, able to quantify and map the static and dynamic pressures sensed, where a higher pressure emitted a brighter field of luminescence. This technology was achieved using an electroluminescent layer with embedded phosphor particles that acted as the dielectric between two stretchable electrodes. A tactile stimulus caused a capacitance change, enhancing the electric field and causing excitation of the luminescent centers of the phosphor particles. By imitating the functions of the mechanoreceptors and nociceptors within biological skin, both gentle tactile and injurious pressure with sensitivities up to 0.66 and 0.044 kPa^−1^, respectively, were distinguished. In 2020, researchers developed an artificial skin that responds to pain [185]. Using bioinspired sensing and neuromorphic engineering technologies, a distinctive microtectonic effect enabled oxygen-deficient, nanopatterned zinc oxide thin films on an elastomer substrate to mimic organic stimulus responses. This may potentially unlock new forms of environmental interactions for robotics and lead to improved rehabilitation medicines.

Though e-skin traditionally compiles information pertaining to vital signs, physiochemical sensing and sampling of fluids, such as sweat, can provide insight into real-time physiological changes that occur at the biomolecular level. In turn, advancing e-skins to take a multimodal approach to data collection will also benefit, and facilitate clinical diagnoses and applications through providing a sustainable surveillance platform with sensing capabilities that are active during regular daily activities. Using a semisolid extrusion-based 3D printing technique, a 2023 study by Song et al. [186] fabricated an epifluidic elastic electronic skin (e^3^-skin) towards the collection of multimodal physiochemical information. As a 3D-printed device, the e^3^-skin is customizable to be manufactured towards user needs, with the implementation of microfluidic channels capable of sampling sweat for analysis, providing information pertaining to glucose intake, pH levels, temperature, and heart rate. Further, the authors implemented the use of machine learning, alongside the metrics of such devices, and results showed that the device was able to accurately predict behavior impairment responses such as reaction time and degree of influence following the participant’s consumption of alcohol.

Finally, e-skins have the additional consideration of requiring a wireless nature as well as flexibility to maximize the comfort and efficacy amongst device users. The use of circuit chips within e-skins thus impedes their intended use alongside their usage of power. To replace the circuitry and components typical of e-skins, a recent development by Kim and colleagues has turned towards the use of surface acoustic wave sensors with piezoelectric gallium nitride (GaN) membranes [187]. Upon mechanical stress, the electrical charge generated by the GaN flexible film allows for communication without a chip. For the optimization of e-skins regarding efficiency, cost, and flexibility, high-sensitivity chip-less solutions hold important value for future work.

### 2.5. Visual Prostheses

Vision loss affects more than 40 million people globally [188]. In vision loss cases involving severe degeneration or damage to the retina, optic nerve, or brain, there are no effective treatments for many visually handicapped people. Today, scientists have succeeded in creating neuroprosthetics able to restore some degree of vision in many visually impaired patients. Acquired blindness is often the result of damage to visual structures such as the retina, optic nerve, or visual processing structures of the brain. For the restoration or improvement of vision in individuals with visual impairment, often, interventions are based upon the intact, remaining structures [189]. As such, potential prosthesis users include those that (1) are not diagnosed with psychiatric disorders that may impact mental status, (2) possess good physical health to lower any potential risks, (3) fit electrophysiological criteria, and (4) fit visual function criteria [190]. The microelectronic device makes functional tissue contact, exciting the neurons at some point beyond the damage site, targeting the retina [191], optic nerve [192], lateral geniculate nucleus [193], and visual cortex [194]. The visual cortex, located in the posterior aspect of the cerebral cortex, is responsible for receiving and processing visual information, often remaining intact in the cases of acquired blindness [195,196]. As such, and under the assumption that visual percepts of small spots of light produced using electrical stimulation via visual cortex devices will combine into coherent percepts of visual forms similar to pixels on a video screen [196], these devices are capable of bypassing damaged structures. Therefore, and by directly stimulating the visual cortex, this technology holds promise for the retrieval of vision through the receival of small amounts of light. Tactile stimulation of the visual cortex in a past work by Beauchamp and colleagues [196] was unsuccessful after taking static approaches, which applied electrical stimulation into a specific form simultaneously. However, the dynamic, path-like stimulation, which traces forms in real time onto the visual cortex, displayed success in enabling the recognition of letters in both sighted and blind participants. Retinal prostheses have been the most successful treatment approach to date and these organic devices have been used to help people recover retinal light sensitivity for retinal degenerative diseases such as retinitis pigmentosa, where the retinal light sensitivity progressively degrades over time [197]. A major goal of the artificial retina is to enhance and restore vision in patients with retinitis pigmentosa (RP). RP is a genetic disease that causes the slow death of rod and cone cells, which are cells that detect light in the eye. In RP, night vision typically declines in childhood, followed by the gradual loss of peripheral vision. By the age of 40, patients usually see the world through a small, central dot, comparable to a straw. Artificial retinas are ground-breaking advancements that could give the gift of vision back to those struggling with RP. The majority of retinal implants are based on inorganic semiconductor chips that create electrical stimuli upon the absorption of photons or the input supplied by an external visor. The world’s first artificial retina, the Argus II^®^, earned FDA approval in 2013. It works by using a small camera attached to glasses sending information from the surroundings to a small device implanted on the retina. In the years since then, scientists have been working hard to develop better and clearer artificial retinas. In addition to the Argus II^®^, the Alpha IMS^®^ and PRIMA^®^ retinal prostheses are devices currently in development. Alpha IMS^®^ is unique in its retinal implant design, as it essentially eliminates the use of glasses, instead using subdermal cables, transmitters, and receivers [197]. It is approved for use in Europe, and FDA approval is expected imminently. The PRIMA^®^ is a silicon-based, wireless retinal prosthesis that utilizes infrared radiation to transmit images from the retina to the optic nerve. It is not yet approved anywhere in the world.

Researchers have identified three distinct configurations of retinal prostheses based on their location in the eye. A suprachoroidal prosthesis is distant from the retina and placed on the periphery of the choroid [197]. Its surgical implantation is simple, and the suprachoroidal prosthesis can stimulate the retina across tissues. A disadvantage of this design is its far distance from retinal neurons, leading to reduced visual acuity. Also, epiretinal prostheses are placed in the retina’s deepest part, in contact with the axon bundle that form the optic nerve and ganglion cells of the retina. An advantage of epiretinal devices is their close proximity to these structures. Minimal currents are needed to transmit neural responses, but patients are usually unable to perceive light as they did before the implant, as the inner retinal circuitry is excluded with a device this deep. This forces patients to become accustomed to a new way of perceiving visual stimuli. Lastly, retinal prostheses may be in a subretinal configuration, in which the prosthesis is found on the peripheral retina between the choroid plexus and degenerated photoreceptors. This location is ideal as the visual physiologic pathway is maintained; however, the positioning of the device is a dangerous surgical procedure and may cause retinal detachment upon insertion.

**Figure 3:**
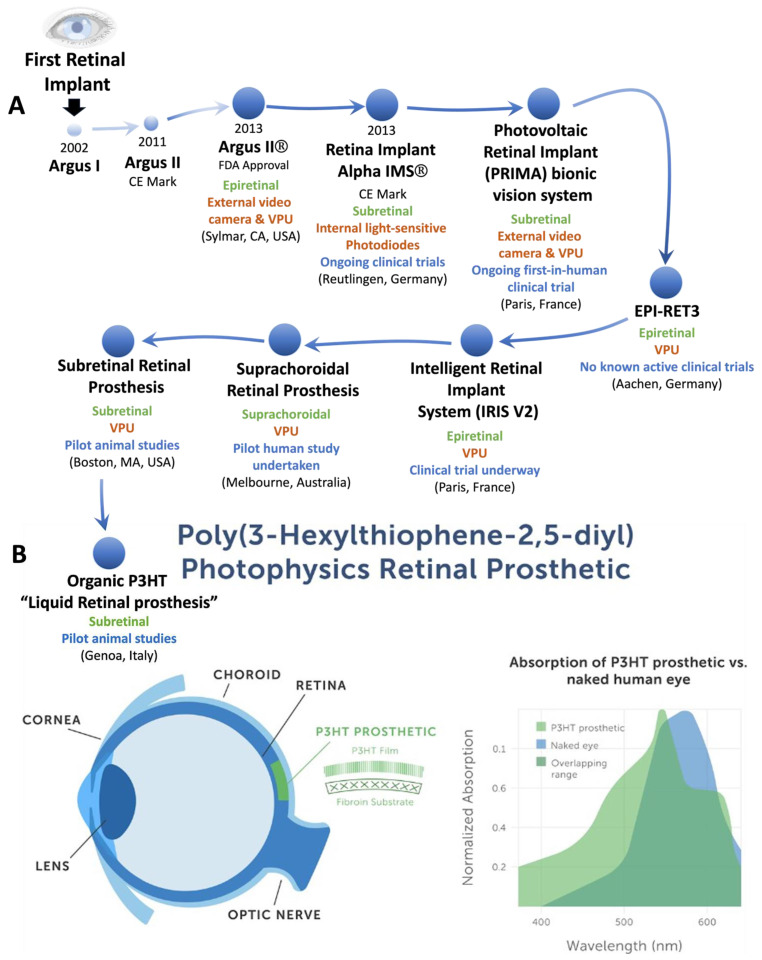
(**A**) The first retinal implant (ARGUS I) was developed in 2002, and later, ARGUS II^®^ received FDA approval in 2013. Presently, Retina Implant Alpha IMS^®^ has undergone clinical trials; the PRIMA bionic vision system, the IRIS V2 and Suprachoroidal Retinal Prosthesis have also been tested in human studies. The EPI-RET3, Subretinal Retinal Prosthesis and the fully organic P3HT prosthesis are more recent devices that have been studied using pre-clinical models only. (**B**) The P3HT retinal polymer prosthetic is biocompatible and can cause strong neural responses in the same way as when naturally responding to impulses from rods or cones. This light-sensitive implant material is able to extend the wavelength over which an animal is able to detect light as well as improve visual acuity in rodents. Although proven to be efficient, the working mechanism is not fully understood [198].

The latest advancement in retinal prostheses is the development of a thin, light-sensitive polymer known as Poly(2-hexylthiophene-2,5-diyl) (P3HT) that is noted to be biocompatible and can cause strong neuron responses, in the same way a neuron would respond to impulses from rods or cones [197] (Figure 3). This subretinal P3HT polymer implant was tested in rodents recently and was found to improve the rodent’s light sensitivities and visual acuities compared to non-implanted rodents [198]. In addition, behavioral responses to light in these rodents were comparable to healthy rats, with records of increased basal metabolic activity of the primary visual cortex in implanted rodents. The Italian Institute of Technology is working on a similar implant with plans to bring the creation to human clinical trials soon.

The telescopic contact lens is another advancing visual technology. Telescopic lenses are those that have miniature zoomable telescopes within that allow users to magnify vision up to 2× through a simple wink. Ford and colleagues have developed scleral contact lenses activated by a 0.5 s wink and allow for refractive correction and 2.8× telescopic magnification [199,200,201]. Although this is an astounding advancement, there are still some significant design challenges with the lens, such as adequate oxygenation to the eye, relearning of proprioception, and visual field movement illusions [202]. Clinically, these lenses seem to be ideal for patients with mild to moderate visual impairment, as they allow magnification only up to 2.5×. Still, the lens may be useful for other ocular diseases like age-related macular degeneration. As this technology continues to develop, the optical advantages of these telescopic contact lenses today include reduced weight, cosmesis, and higher magnification levels.

## 3. Augmented Human Performance

### 3.1. Biohacking and Prosthetics for Human Enhancement

**Figure 4:**
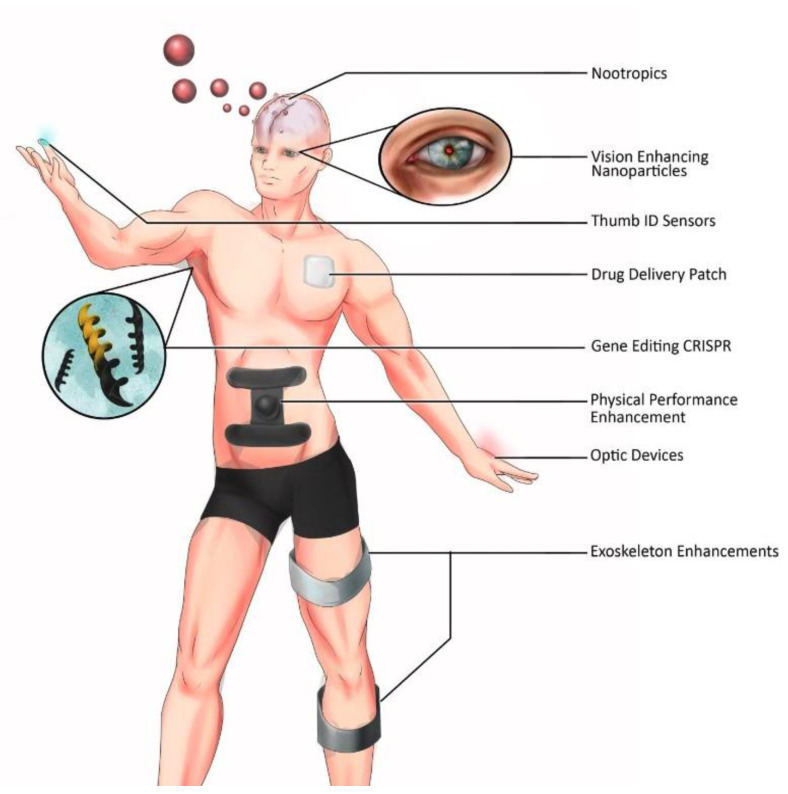
Biohacking can be as simple as changing your diet, listening to music, and taking supplements, to trying to change your gut microbiome, gene therapy, or methods to modify genetic or brain function to improve one’s self (faster, stronger, mitigating a predisposition for a disease, better focus, memory, energy, etc.) It also refers to devices that may extend or improve human capabilities to enhance the human condition such as exoskeletons and implantable biosensors.

Inspired by transhumanism concepts, self-experimentalists have inserted commercial or homemade implants under their skin, via hypodermic needles or following surgical incision where implants are protectively coated and thus bioproofed using medical-grade borosilicate glass capsules, Parylene C, polytetrafluoroethylene, titanium nitride, or silicone, for example, to attenuate an immune response [203]. Some intended uses include human–electronic device communication, self-quantification to maintain a healthy lifestyle, as well as cosmetic enhancements [204] (Figure 4). While much of this type of augmentation has been conducted by self-experimentalists, significant academic work has also been conducted to explore augmenting human perceptive senses and the human–machine interface. Body modification practice is reported as far back as 2004, where a multielectrode array was implanted into the median nerve in the upper limb of a volunteer to establish the bi-directional interface between the human nervous system and a computer. The aim was to assess the efficacy, compatibility, and long-term operability of the BMI which controlled a robotic arm and connection via a remote system with another human implantee via the internet [205]. Real-time and remote control of the instrumented prosthetic hand was achieved using this bi-directional link. Enabling interactivity between humans and technology may also have a convenience component in addition to the therapeutic outcomes. Privacy, ethical, and practical issues arise from current implementation. The technology company Three Square Market, for example, has developed a microchip that can be implanted between the thumb and forefinger of its volunteer employees, who are now able to use and pay for the vending machine snacks with the swipe of a hand [206,207]. The vending machine immediately deducts money from the volunteer’s bank account. Radiofrequency identification (RFID) tags have also been tested in Sweden, where subcutaneous microchips are being used to allow people to enter secure buildings or book train tickets [208].

Other body modification examples include the sub-dermal insertion of neodymium magnets to mimic magnetoreception. About 50 species including homing pigeons, mammals, reptiles, fish, and bats use magnetoreception for sensing orientation and navigation, although the mechanisms that underlie the magnetic compass that responds to the Earth’s approximately 50 µT fields are still poorly understood [209]. Inspired by magnetoreception and prior to their implantation into the finger, each neodymium magnet was coated with titanium nitrate and discs measured 3 × 1 mm in size. The magnets allowed for the increased ability to perceive an electromagnetic stimulus via tactile sensation where less force was needed in seven implanted humans when compared with the control group [203]. Similarly, magnets have been implanted into the tragus of the ear with reported delivery of audio signals to the ear from a coil of electromagnetic wire connected to a smartphone. Silicon-coated electronic implants designed to connect people with the earth’s magnetic field and inserted within the subdermal layer of the chest are reported to vibrate when the recipient faces north (Cyborg Nest). This artificial sensing technology includes a miniaturized compass chip and Bluetooth connection, attached to the skin using two titanium bars similar to a piercing [210]. Biohackers have also inserted two near-field communication chips into the fingers to remotely link to websites or open car doors among other tasks. Temperature sensors developed for veterinary use have been utilized to measure human body temperature in real time and within a detection range of 25–43 °C. Using a hypodermic needle, coated sensors were implanted into the arm close to the armpit and was able to transfer data to a tablet computer via a Bluetooth connection [203]. Optical materials and devices that utilize the decay of tritium gas to produce radioluminescence of devices featuring light-emitting diodes have also been implanted subcutaneously for cosmetic reasons.

Finally, our visual system detects light between 400 and 700 nm (visible light) and mammals cannot see light over 700 nm in wavelength due to limitations in the physical thermodynamic properties of the photon-detecting opsins. In 2019, a study by Ma et al. [211] developed ocular injectable photoreceptor-binding upconversion nanoparticle antennae that allowed animals to detect longer wavelength light, such as near-infrared (NIR) light. This successful integration of self-powered, biocompatible nanoparticles with biological systems has developed abilities that do not exist naturally by extending vision and allowing mammals to “see in the dark”. These implanted nanoantennae did not interfere with normal visible light vision and animals were able to detect NIR and visible light images simultaneously. However, it is important to note that in this device-related context, biohacking is a niche area where wide-spread acceptance, a large body of participants and information on any long-term side-effects are currently lacking, making results and questions surrounding enhancement success and sustainability mostly speculative.

### 3.2. Genetic Engineering Technology

Although not a device, the review would be incomplete if it did not include genetic engineering technology, which is at the forefront of medical research advancement. These technologies allow genetic material to be added, removed, or altered at specific locations in the genome. In particular, the use of CRISPR-Cas9 (clustered regularly interspersed short palindromic repeats)-(CRISPR-associated protein 9) technology is of high significance as it is a simple, yet powerful tool used to edit genomes, the elements that contain all the genetic material of an organism. CRISPR technology was initially adapted from the natural defense mechanisms of bacteria, a process where microbes cut up and destroy the DNA of a foreign invader. To date, this biomimetic technology allows researchers to easily alter DNA sequences and modify gene function, with conceivably numerous potential applications, including correcting genetic defects and in treating and preventing disease [212]. CRISPRs are specialized sequences of DNA, and the protein Cas9 is an enzyme able to act like a pair of molecular scissors, capable of cutting DNA at site-specific locations [213]. The CRISPR regions have two distinct characteristics: the presence of nucleotide repeats and spacers. Repeated sequences of nucleotides, the building blocks of DNA, are distributed throughout the CRISPR region. The spacers are segments of DNA that are interspersed among these repeated sequences. In the case of bacteria, the spacers are taken from viruses that previously attacked the organism and serve as memory banks, enabling the bacteria to recognize the virus in the future.

The Cas9 protein typically binds to two RNA molecules, the CRISPR RNA (crRNA) and transactivating crRNA (tracrRNA). The crRNA bases pair to the tracrRNA to form hairpin loops, guiding Cas9 to the target site, where specific strands of double helix DNA are cleaved [212,213,214,215]. However, cleavage only occurs if a “protospacer adjacent motif” (PAM) DNA sequence is present near and adjacent to the target DNA sequence. Therefore, the PAM sequence acts as a built-in safety mechanism, as the absence of a PAM renders the Cas9 complex unresponsive, ensuring that Cas9 only cleaves at specific DNA sites. The genome encodes a series of instructions within their DNA sequences and editing these genomes will change these sequences and subsequently their function. Any desired changes to the genetic material can be introduced by inserting a cut or break in the DNA and then tricking the cell’s natural ability to repair. DNA strand repair occurs through activation of either non-homologous end-joining (NHEJ) or homology-directed repair (HDR) [213]. Non-homologous repair of the two ends generates insertions or deletions (indels) that result in protein loss and termination of protein translation, disabling the gene [216,217]. In contrast, a break repaired by homologous joining occurs by filling the “gap” in DNA with a sequence of nucleotides (a homologous template). Researchers supply the DNA template of their choosing, thereby writing-in any gene and its subsequent function. NHEJ is especially useful for augmenting loss of function events, and HDR for gene function recovery [212].

CRISPR-Cas9 technology and the use of this precision medicine approach has a multitude of potential current and future clinical applications. For example, more than 98% of the human genome does not code for proteins and evidence suggests that many noncoding regions have functional elements that directly impact gene regulation and disease and are therefore critical for human health [218]. CRISPR-Cas9 could be used to perform wide-scale genomic screening to identify noncoding oncogene regions, sequences that contribute to the development of cancer, for example, or noncoded regions that contain functional elements involved in chemotherapy drug resistance [219,220]. This could significantly impact our success not only in the prevention and treatment of cancer, but in other single-gene disorders such as cystic fibrosis, hemophilia, and sickle cell disease, hereditary diseases as well as complex diseases such heart disease, mental illness, and infectious disorders including human immunodeficiency virus (HIV) [221]. Further, genetically engineered bacteria and other microorganisms are currently used to mass produce human insulin, human growth hormone, human albumin, monoclonal antibodies, antihemophilic factors, and other pharmaceuticals. The number of such compounds is projected to increase into the future [222]. Regarding cancer, the first clinical trial in 2016 used CRISPR-Cas9 to create a PD1 knock-out in chimeric antigen receptor (CAR) T cells for the treatment of bladder cancer, meta-static renal cancer, and non-small cell lung cancer. This trial demonstrated significant clinical improvements but also reported negative adverse events such as cytotoxicity and neurotoxicity [223]. But not all experiments fell short; the FDA has already approved two CAR T cell products that target CD19 to effectively treat acute lymphoblastic leukemia and non-Hodgkin Lymphoma [221]. The FDA has also approved the use of genetic engineering to treat patients with Leber congenital amaurosis [224]. Duchenne’s Muscular Dystrophy (DMD) is an irreversible and progressively severe inherited disorder that has distinctive exon characteristics that allow gene editing using NHEJ repair following DNA cleavage [213]. DMD is characterized by reduced levels of dystrophin, where muscle cells become damaged and weaken over time. Using CRISPR-Cas9 technology, studies suggested that exon skipping could restore levels of dystrophin function because most exons implicated in the disease contain a multiple of three bases, and the severity of presentation may become reduced if these exons are skipped [225]. Several mouse studies demonstrated restoration of a significant level of dystrophin function via exon skipping [226,227,228] with the use of NHEJ repair techniques.

Further, there are many important opportunities for the use of deep learning and neural networks to improve modern genomic technologies, which will advance our understanding of the genetics of disease and ultimately help clinicians provide more accurate treatments and diagnoses. Algorithms that scale to very large patient cohorts can be used to seek and discover causal genetic mutations by correlating their expression to specific traits or medical outcomes. Such algorithms could discover previously unseen relationships to disease as well as interpret data and predict the effects of genetic mutations on disease risk or drug response, among many other elements. However, any new technology takes some time to understand and perfect and although an extremely powerful tool, this technology presently has important limitations. CRISPR-Cas9 can be implanted into humans; however, it is difficult to deliver in large numbers and presently, the most common method of delivery is through use of nonintegrating adeno-associated virus and integrase-deficient lentivirus vectors. The use of viral vectors remains a problem for translation into many clinical applications [218]. Additionally, CRISPR-Cas9 is not 100% efficient or accurate as cells following uptake sometimes do not exhibit genome editing activity or can produce “off-target” edits. Nevertheless, the CRISPR-Cas9 system has generated a lot of excitement in the scientific community because it is faster, cheaper, more accurate, and more efficient than other existing genome methods and there is no doubt that it has become a valuable tool in medical research.

Lastly, CRISPR/Cas9 may be used to modify human embryos using induced pluripotent stem cells and hematopoietic stem cells. Several research groups have demonstrated the use of this technology in human zygotes to prevent inheritable diseases [229,230]. Advances have been shown in the treatment of Hemophilia B, Beta hemoglobinopathies, cardiac conditions, and other genetic diseases [221]. The discussion of CRISPR-Cas9 for the use of phenotype alterations in human zygotes is of interest but brings forth ethical inquiries. How far is too far? Should parents be able to alter their child’s eye and hair color or height for example? Any such edits would not only affect an individual, but also his or her progeny. Will this technology only be available to those of higher income? Will the implementation of such genetic modification turn to augmentation and lead to a growing divide in human physical performance, cognition, or even life expectancy for small segments of society? In all, CRISPR-Cas9 technologies have made marked advancements over the past decade, but further scientific progress and issues involving ethics need to be discussed, particularly the ethical implications in altering embryotic genomes.

## 4. The Ethical Challenges of Augmented Human Performance

**Figure 5:**
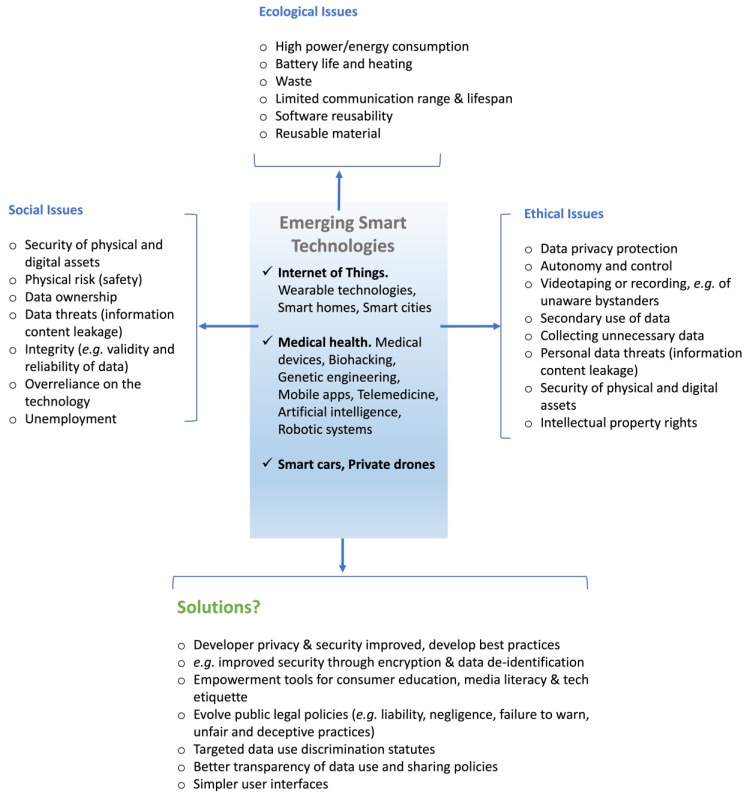
The promise of implantable, wearable, and genetic technology for improving human health, wellbeing, and performance has never been greater. However, novel smart technologies that are able to biointerface with the body and/or society, raises social, ethical, and environmental issues. As this field continues to progress and their use becomes increasingly inseparable from the human world, the implementation of strategies that address these issues are needed.

Questions regarding the ethics of rapid implementation and development of such devices and technologies abound, especially with the promise of applications for therapeutics, rehabilitation, and augmentation of historically understood human performance limits (Figure 5). From implements to interfaces, these devices will restore lost functionality for the medically vulnerable and have the potential to augment new strengths and resilience in healthy others. Generally, electronic devices are expected to take over almost every aspect of our lives and are becoming increasingly inseparable from the human world.

A glimpse into the future of electronics suggests a trend towards a hybrid of technology and consumer demands, with society prioritizing progressively futuristic ideals of new and enhanced consumer experiences. This is forecast to include a series of high-end technologies such as robotic systems with human-like sensing capabilities, smart wearables (i.e., tech gadgets/devices integrated into clothing or human body), autonomous (i.e., self-driving) cars, non-invasive health monitoring and critical care, and radio-frequency identification tags [167,169,178,231]. These devices blur the line between the digital and the physical, making them difficult to distinguish. The merging of digital and physical interactions has consequences for both, while our society’s adoption of new mixed reality sensors and tools continues unabated.

The right-to-repair movement, formally started in 2018, is characterized by the notion that individuals should own products that they have purchased, including the freedom to choose who can repair them [232,233]. This political movement revolves around improving consumer-centric business practice, preventing manufacturer monopoly and reducing overall electronics-based waste [233]. After a medical device company shuttered their retinal implant prosthesis, users of the devices found themselves in uncertainty, unable to access repairs if the device malfunctioned [234]. Though users of the retinal implant have since received support following company acquisition, poor customer support could have been avoided. Similarly, a dental aligner company left customers uncertain in their next steps following bankruptcy [235]. Consumers were then directed to local dentists and orthodontists for further support, despite the company’s initial intention to avoid office visits [235]. Whether due to product line discontinuation or corporate sale, medical devices or human augmentations necessitate long duration product life-cycle planning. All devices, whether used in the context of rehabilitation or not, are likely to require repair or replacement within their lifespan. Prior planning of devices used for human augmentation is critical, as the irreversible nature of the technology requires consistent access in the case that repair is needed. Guidance and best practices developed in response to mobile phone repair may be appropriate to carry forward to medical devices, where monopolization of repair efforts hinder consumer experience. In the United States, Executive Order 14036 signed in 2021 encouraged the Federal Trade Commission to exercise rulemaking authority with a focus on enabling third party repair [236].

The right-to-repair movement seeks to address consumer issues in a wide array of technological fields through legislation. The European Commission has proposed initiatives to protect consumer rights, aiding in the reduction of waste and supporting the objectives of the European Green Deal [237]. In the United States, Congress has proposed the Fair Repair Act, which is a bill requiring manufacturers to provide the necessary information, tools, and components for diagnostics of repair [238]. However, further progress has yet to be made at this time. On the state level, progress varies, as 33 states alongside Puerto Rico considered right-to-repair legislation [239]. According to the Repair Association, an organization supporting the movement, California [240], Colorado [241], Massachusetts [242], Minnesota [243], and New York [244] have passed laws to provide more repair options to citizens. Though these laws do not particularly apply to medical devices in specific, future progress may result in such technologies being included. He et al. [245] states that the right-to-repair medical equipment would provide a solution to manufacturer-dependent repair that may have proven useful during the COVID-19 pandemic. Implications of unrestrained access to repair and manufacturing information in neuroprosthetic development poses a new, unprecedented challenge in device development.

As society continues to press toward a more environmentally sustainable future, a “green” approach is increasingly important. Device manufacturers must meet sustainability requirements involved in their advanced manufacturing, and as they create next-generation wearable, implantable, self-resorbable electronics and energy-devices. As the lifetime of electronic devices becomes shorter and shorter, the pressure on e-waste management systems is mounting with no end in sight [246]. The technology transformation will require our society to curb the surging amounts of electronic waste (e-waste) generated [246,247], which poses severe environmental and health concerns worldwide. Astonishingly, almost 60 million kilograms of e-waste is generated every year even in a small city-state like Singapore [246], which exceeds the combined weight of approximately two hundred and twenty Airbus A-380 aircraft. Such “green” electronics and energy devices will also be highly applicable for next-generation applications (e.g., artificial intelligence-driven cosmetics, skin-like electronic bandages) and are of utmost importance especially for advanced biomedical or related biological applications [248].

Continued conversations about proper ethical approaches for both the adoption and implementation of these classes of disruptive technology are necessary and this is perhaps the best opportunity to include diverse voices and refrain from marginalized affected groups. Within the field of transhumanism, there remains different perspectives and attitudes about what it means to be human and the theory of change [247]. The important ethical concepts considered by Lee [249] of (i) autonomy, (ii) identity, (iii) futures, and (iv) community were discussed around the topic of cochlear neuroprosthetic implants and the reception by the deaf community. Cochlear implants have exceeded the initial predictions about consumer (patient) acceptance and have become the most successful neural prostheses to date [250], though there is variation in outcomes between unilateral and bilateral implementation [251]. However, as global healthcare coverage policies vary, health insurance companies in certain areas may deem these devices or their bilateral implementation as having a limited benefit and thus not subject to reimbursement. This situation provides a unique lens for framing discussions of the next generation of implants and augmentations. For example, while an effective tool, members of the deaf community did not have uniformly positive responses in their welcoming of the device due to what was viewed as experimental or unsound science and ethics [252]. Accessibility both in the prescription of devices and their financial coverage will remain key components to equitable implementations of new human augmentation technologies, including for rehabilitation devices.

As seen through Lee’s lens [249], key ethical issues can be applied retrospectively to cochlear implant implementation, which provide insights on the framework of health and social justice, as well as ethical ways forward for new technologies that can potentially transform rehabilitation, therapeutics, or advance physical augmentation. These key ethical concepts posed by Lee include the following:

1. Autonomy: Free individuals in a forthcoming enhanced, transhuman, and posthuman society, containing social norms and anticipated experiences of community pressure.
2. Identity: Technologically-altered or alterable human nature, its dignity, normality, with choices of elective enhancements and elective disability (and disadvantage).
3. Futures: Children’s welfare, parents’ preferences but with obligations to their children’s future living in society.
4. Community: Government and society’s responsibilities to diverse groups; integration, exclusion, and fears of extinction of minorities.

When considering new rehabilitative or augmentative devices, (including implantable neuroprosthetics with artificial intelligence to amplify cognition), these four questions can begin a conversation about medical ethics. Discussions including privacy and user autonomy concerns, security of the device, access to repairs, and potential health and behavior risks are all valuable considerations. Ideally, these discussions should be conducted prior to device development, patient acceptance, and adoption [253], as public policy tends to lag behind technological implementation. Other devices may allow for incredible gains in rehabilitation, restoring function, and activity to those in vulnerable medical states. As similar technology may allow for cosmetic changes, such as changing body presentation including eye color, or potentially increasing muscle mass, strength, or height prior to birth, current policy debates are centered around medical rehabilitation versus physical augmentation. As the large data sets that these devices generate begin to be aggregated by corporations whose interests may include improving their applications or generating more targeted advertisements, questions will likely include the following: “What mechanisms are in place to prevent data hacking?” “Who has a right to users’ data?” “Who has access to repair technology?” and “What will be considered protected ‘health’ data as the lines between wellbeing and augmentation blur?”

## 5. Continued Discussion

Recent contemporary scientific development has established the critical role that technological advancement has in driving the future of healthcare as many biomedical discoveries have contributed to lifesaving and life-changing devices and treatments that restore health. This exciting revolution is currently driving the pace where treatments are becoming smaller, faster, and smarter and the multi-faceted approach of combining the fields of materials science, engineering, and medicine is spearheading these clinical breakthroughs. No longer are these materials inert substitutes that simply replace tissue, but increasingly, the new implant is engineered to interface with the body in a deliberate and dynamic way. Smart macro-, micro-, and nanoscale strategies involving the use of newer biomaterials that are chemoresponsive, electroactive, photomechanical, piezoelectric, temperature responsive, thermoelectric, pH-sensitive, or contain shape-memory behavior will undoubtedly contribute to novel technological endeavors. As such, the promise of implantable and wearable technology for improving human health, wellbeing, and performance has never been greater. However, questions remain surrounding its implementation, diffusion, and security, including “Will the technology diffusion curve and rapid advancement create distinct subcultures, or will we first see the end of debilitating disease symptoms?” and “What are the processes in place to ensure equitable availability to the technology?”

Availability to repair hardware and software for biomedical devices remains at the ethical forefront of debate [245,254]. The legality of right-to-repair continues to change across a variety of industries, as state and federal governance alter stances on the issue. As technology continues to develop and progress, these concerns will only develop and grow with them. Intellectual property rights, monopolization concerns, and financial cost among other factors should be considered in the development of opinions. Though there are complexities attributed to choosing a definitive stance in the manufacturing of a device and development of a company, future planning is crucial for the benefit of all parties. Regulatory intervention may need to be discussed to develop procedures, in case of company closure or sale, should users of devices lose access to maintenance or repair. At this time, the field is experiencing early challenges with device availability [234,235]. Public policy is still developing in regard to the adoption and extent that right-to-repair plays a role within the world of device manufacturing across multiple disciplines [238]. It is now critical to consider the implications of severed service for users who rely on their devices for life support or daily activity, where a disruption to a device’s repair or maintenance would potentially require additional surgery.

## 6. Conclusions

The convergence of miniaturized electronics, advanced biocompatible materials, and the rise in transdisciplinary research has the potential to rapidly change the medical industry and our understanding of a world with a near seamless human–machine interface. Brain–machine interfaces, neuroprosthetics, exoskeletons, implantable/wearable devices, and genetic engineering all have the potential to expand our human race beyond normal physical and cognitive limitations. Nevertheless, significant technological challenges remain. To achieve the maximal benefits offered by BMIs and prosthesis control as well as optimal exoskeleton functionality while also permitting effective user control, sophisticated technological improvements are needed. For example, the device and their respective control system/s must accurately follow and augment the user’s movement when challenged under diverse scenarios. In these situations, there is a critical need to decode user intent in real time, thereby responding, reconstructing, and delivering precisely controlled movement patterns that provide safe and “real life” functionality. In this respect, and due to the complexity of natural human motor capabilities, existing devices remain relatively limited. Further, effective sensory feedback into the sensory cortices within the brain is required as it is essential for bidirectional information flow. This too remains relatively limited and further advancements are vital to facilitate future next steps towards enhanced sensorimotor performance, function, and device learning. Future discovery and innovations in actuation, sensing, control, and neural interface technologies parallel with an improved understanding of neuromuscular principles will continue to evolve this technology. Furthermore, continued advancements in sensory and machine-learning technologies in combination with enhanced materials that are durable and lightweight will also propel these fields forward, potentially leading to a substantial decrease in healthcare expenditures. Similarly, continuous evolutions and increments in the development of deep learning and neural networks will undoubtedly advance genetic engineering, further driving and accelerating our understanding of genetics and disease. Nevertheless, ongoing development will also deliver anticipated gains and opportunities towards human augmentation. As such, and as these extensive research pursuits expeditiously progress, human augmentation may become commonplace sooner than we expect, possibly resulting in a growing divide in human physical performance and cognition for those who use it. Importantly, now is the time for continued discussion of the ethical strategies for research and implementation as well as deciphering and assembling a long-term approach that enables device repair and replacement. Together, these approaches will be fundamental for the future use of this technology either in the context of human rehabilitation or in augmentation. 

## Data Availability

Data available within the article.
